# Mesenchymal Stem Cells and Atopic Dermatitis: A Review

**DOI:** 10.3389/fcell.2020.00326

**Published:** 2020-05-14

**Authors:** Sérgio Ricardo Teixeira Daltro, Cássio Santana Meira, Ivanilson Pimenta Santos, Ricardo Ribeiro dos Santos, Milena Botelho Pereira Soares

**Affiliations:** ^1^Gonçalo Moniz Institute, Oswaldo Cruz Foundation (FIOCRUZ), Salvador, Brazil; ^2^Health Institute of Technology, National Industrial Learning Service - Integrated Manufacturing and Technology Campus (SENAI-CIMATEC), Salvador, Brazil; ^3^National Institute of Science and Technology for Regenerative Medicine (INCT-REGENERA), Rio de Janeiro, Brazil

**Keywords:** mesenchymal stem/stromal cells, atopic dermatitis, atopic eczema, immunomodulation, inflammatory skin diseases

## Abstract

Mesenchymal stem/stromal cells (MSCs) are stromal-derived non-hematopoietic progenitor cells that reside in and can be expanded from various tissues sources of adult and neonatal origin, such as the bone marrow, umbilical cord, umbilical cord blood, adipose tissue, amniotic fluid, placenta, dental pulp and skin. The discovery of the immunosuppressing action of MSCs on T cells has opened new perspectives for their use as a therapeutic agent for immune-mediated disorders, including allergies. Atopic dermatitis (AD), a chronic and relapsing skin disorder that affects up to 20% of children and up to 3% of adults worldwide, is characterized by pruritic eczematous lesions, impaired cutaneous barrier function, Th2 type immune hyperactivation and, frequently, elevation of serum immunoglobulin E levels. Although, in the dermatology field, the application of MSCs as a therapeutic agent was initiated using the concept of cell replacement for skin defects and wound healing, accumulating evidence have shown that MSC-mediated immunomodulation can be applicable to the treatment of inflammatory/allergic skin disorders. Here we reviewed the pre-clinical and clinical studies and possible biological mechanisms of MSCs as a therapeutic tool for the treatment of atopic dermatitis.

## Introduction

Atopic dermatitis (AD), commonly known as atopic eczema, is a typical dermal chronic inflammatory disorder characterized by eczematous cutaneous lesions and severe pruritus, representing a significant burden on health-care resources and patients’ quality of life (Hoare et al., 2000; Boothe et al., 2017). The prevalence of AD is higher than 20% in children and 1–10% of adults in some countries and continues to increase, affecting not only low-income, but also developed countries (Nutten, 2015; Reed and Blaiss, 2018; Sacotte and Silverberg, 2018).

AD is considered the most expensive cutaneous disorder in the world, followed by acne and psoriasis (Sacotte and Silverberg, 2018). The treatment of AD is based on pharmacological intervention, through the use of corticosteroids, calcineurin inhibitors, leukotriene receptor antagonists and antihistamines (Nakagawa et al., 1994; Berth-Jones et al., 2003; Ring et al., 2012a, b). The use of these classes of drugs, however, not only provides temporary relief of symptoms, but also causes various adverse effects and drug resistance in long-term treatment (Saeki et al., 2016; Silverberg and Durán-McKinster, 2017). Therefore, the development of safe and effective therapies is necessary for the proper management of patients with AD.

Mesenchymal stem/stromal cells (MSCs), the most common stem cell used in cell therapy field, are multipotent, undifferentiated and self-renewing cells found in many adult and neonatal tissues (Pittenger et al., 1999; Dominici et al., 2006; Wei et al., 2013). MSCs have tissue repair potential through their self-renewal and differentiation abilities and is considered a strong modulator of the immune system through modulation of proliferation, recruitment and function of immune cells from innate and adaptive immune system (Herrero and Pérez-Simón, 2010; Hypnes et al., 2016; Zheng et al., 2018; Poggi and Zocchi, 2019). These features boosted pre-clinical and clinical investigations with the purpose of evaluate MSCs on autoimmune and immune-related diseases, such as asthma, systemic lupus erythematosus, rheumatoid arthritis, multiple sclerosis and skin diseases, such as AD (Sun et al., 2009; Nemeth et al., 2010; Wang et al., 2013; Na et al., 2014; Shin et al., 2017a; Li et al., 2020). In this review, we provide an overview of current reports regarding the use of MSCs as a therapeutic tool in atopic dermatitis, the challenges of conducting MSC studies in AD, as well as future directions needed to develop this field.

## Atopic Dermatitis

AD is characterized by severe itchiness, being one of the most common chronic inflammatory skin diseases, affecting up to one-fifth of the population in developed countries (Weidinger and Novak, 2016). AD can occur in any age group or ethnicity, being the most common skin disease in children, especially infants under two years of age. In some countries, especially in Asia and Latin America, it affects around 20% of children (Nagaraja et al., 1996; Odhiambo et al., 2009; Nutten, 2015; Page et al., 2016). Although most cases of childhood AD spontaneously resolve by adulthood, the disease persists in 10–30% of cases (Ellis et al., 2012). More rarely, the first symptoms develop in adulthood, being the prevalence of AD in adults of ~1–3% (Eichenfield et al., 2014; Nutten, 2015).

**FIGURE 1:**
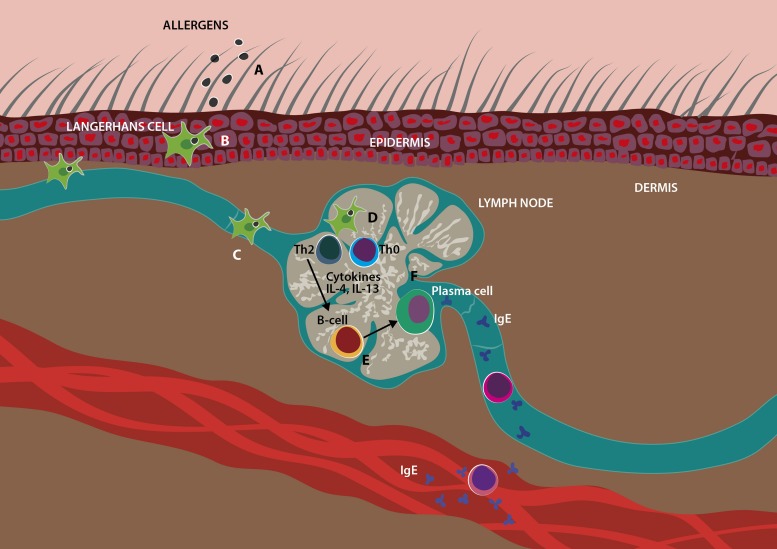
Skin barrier dysfunction undergoing sensitization in AD. Allergens penetrating the skin **(A)** can be captured by Langerhans cells, which migrate to a regional draining lymph nodes **(B,C)**. Langerhans cells present the allergen to a naïve T cells, causing polarization to a Th-2 phenotype which secrete cytokines, such as IL-4 and IL-13 **(D)**, that stimulate B cells to become plasma cells and secrete allergen-specific IgE **(E,F)**.

**FIGURE 2:**
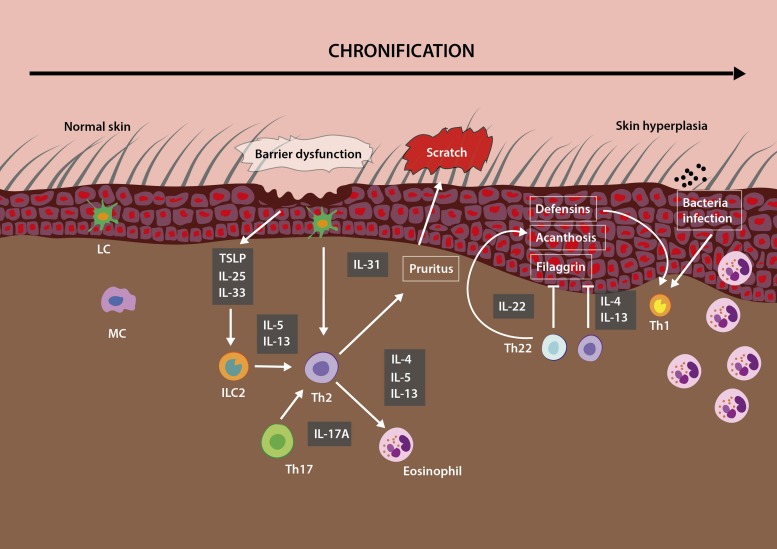
Immune responses involved in chronification of lesions in AD. Scratching resulting from pruritus (the main symptom of AD), makes keratinocytes react by releasing cytokines important for inflammation, including TSLP (thymic stromal lymphopoetin), IL-33, and IL-25. IL-33 activates innate lymphoid cells 2 (ILC2) and Th2 lymphocytes. The release of IL-31 stimulates even more the pruritus. In response to IL-22, keratinocytes proliferate, resulting in diffuse epidermal hyperplasia. Eosinophils accumulate in chronic atopic skin. MC: mast cells; LC: Langerhans cells; ILC2: Innate lymphoid cell 2.

The pathogenesis of AD is multifactorial, including genetic factors, epidermal barrier defects and immunopathogenic factors (Meagher et al., 2002). Regarding the immunopathogenic abnormalities, lymphocytes play a critical role, since T helper 2 (Th2) lymphocyte activation and the cytokines released by them lead to elevated production of immunoglobulin E (IgE) (Figure 1), increased inflammation in the skin, and aggravate the skin barrier defect in AD (Meagher et al., 2002; Klonowska et al., 2018). The initial course of AD is characterized by a biphasic inflammation, where a Th2 profile predominates, with increased levels of several cytokines, including interleukin-4 (IL-4), IL-13, IL-17, IL-22, IL-31, and thymic stromal lymphopoietin (TSLP) (Olivry et al., 2016; Chaudhary et al., 2019). This immune signature exists in lesional and non-lesional skin, indicating a systemic switch to a Th2 profile. In chronic AD skin lesions, a Th1/Th0 dominance has been described with increased production of interferon-gamma (IFN-γ), IL-6, IL-12 and granulocyte-macrophage colony-stimulating factor (GM-CSF) (Figure 2). In addition to cytokines produced from Th1 and Th2 cells, IL-17, produced from Th17 cells, has been reported to contribute to pathogenesis of AD. Interleukin-17 stimulates the production of IL-6 and IL-8 by human keratinocytes, which lead to increased T cell migration to the skin (Nutten, 2015; Miossec and Kolls, 2012; Sidburry and Khorsand, 2017; Campione et al., 2020). Interestingly, MSCs derived from skin samples from patients with chronic AD contributes to pathogenesis of disease through production of several Th1/Th17 cytokines and chemokines, such as CCL2, CCL20, CXCL2, CXCL5, IL-6, IL-8, IL-12, IL-17A, IL-21, transforming growth factor β (TGF-β) and IFN-γ (Orciani et al., 2017).

The clinical manifestations of AD vary with age. Infants (0–2 years) typically present erythematous papules and vesicles on the cheeks, forehead, neck and scalp, whereas children (2 years of age to puberty) exhibit dry skin and lichenified papules and plaques in flexural areas of the limbs. In adulthood, the predominant areas of eczema are the flexural folds, the face and neck, the upper arms and back, hands, feet, fingers and toes (McKenna and Doward, 2008; Weidinger and Novak, 2016; Kapur et al., 2018). Regardless of age, AD affects the patients’ quality of life, once implies in sleep deprivation, financial costs and employment loss (Carroll et al., 2005; Lewis-Jones, 2006; Nutten, 2015).

Currently, there is no cure or prevention technique for AD and, therefore, treatment focuses on disease progression control and alleviation of symptoms (Carr, 2013). Due to the multifactorial pathogenetic mechanisms of AD, a single therapeutic approach is hardly capable of achieving disease control. Topical treatment with moisturizers, emollients or corticosteroids, oral therapy with immunomodulators (corticosteroids, calcineurin inhibitors, leukotriene receptor antagonists and antihistamines) and antibiotics have been the basis for the management of atopic dermatitis (Ring et al., 2012a, b; Aoki et al., 2019). In general, topical treatment is the first option of choice, being effective for the most patients with mild disease. Systemic therapy may be offered to those with severe disease or treatment-resistant eczema, however, invariably, in the long term, comes along with side effects (Apfelbacher et al., 2013; Wong et al., 2017; Aoki et al., 2019). New therapeutic approaches, such as monoclonal antibodies, are passing through the drug discovery pipeline and may reinforce the therapeutic arsenal against AD in a near future (Weidinger and Novak, 2016; Snast et al., 2018; Pistone et al., 2019).

## MSCs Characteristics and Immunomodulatory Properties

MSCs were first described in the 70’s as a cell population with the ability of self-renewing and as precursor cells of osteogenic lineage (Friedenstein et al., 1970). Since then, MSCs have been the target of numerous investigations and, due to their therapeutic potential, it is the most studied stem cell type, with more than 1,000 clinical trials, at various phases, recorded in the clinical trials database (clinicaltrials.gov as of January 2020). MSCs have a potential for differentiating into a variety of mesenchymal cell types, including adipocytes, chondrocytes, myoblasts and osteoblasts (Chamberlain et al., 2007; Glenn and Whartenby, 2014). MSCs are a heterogeneous population of cells that can be obtained by different sources, such as adipose tissue, amniotic fluid, bone marrow, dental pulp, skin, placenta, umbilical cord, and umbilical cord blood (Bianco et al., 2001; Lee et al., 2004; Lu et al., 2006; Guimarães et al., 2011; Antoniadou and David, 2016; Loukogeorgakis and De Coppi, 2017). Due to the pleiotropic nature of MSCs, the International Society for Cellular Therapy (ISCT) listed the minimum criteria and markers of MSCs in 2006 (Dominici et al., 2006). Briefly, MSCs should have plastic-adherence when maintained in standard culture conditions, capacity to *in vitro* differentiation into three cells types (adipocytes, chondrocytes and osteoblasts) and positive expression of specific cell surface markers, such as CD73, CD90 and CD105, while being negative to markers such as CD11b, CD14, CD19, CD34, CD45, CD79, and human leukocyte antigen – DR isotype (HLA-DR) (Dominici et al., 2006; Boxall and Jones, 2012).

MSCs emerged as an attractive cell type for the treatment of a variety of diseases, mainly injured tissues and immune-mediated diseases, due to its ability in modulate innate and adaptive immune system (Wei et al., 2013; Glenn and Whartenby, 2014; Golchin et al., 2019). In the innate immune system, MSCs are able to promote macrophage polarization to M2 phenotype (Kim and Hematti, 2009), inhibit the release of antimicrobial products by neutrophils (Raffaghello et al., 2008), suppress degranulation and production of tumor necrosis factor alpha (TNF-α) by mast cells (MC) (Brown et al., 2011), inhibit natural killer cells (NK) activation and production of pro-inflammatory cytokines (Sotiropoulou et al., 2006), and affect dendritic cell (DC) maturation, cytokine secretion and migration to lymph nodes (Chiesa et al., 2011). Regarding the adaptive immune system, MSCs inhibit B cell proliferation and affect antibodies production (Corcione et al., 2005), and, most importantly, affects T cell function, by inhibiting T cell proliferation through arresting at G0/G1 cell cycle phase, suppressing the development of Th1 and Th17 cells and favoring the development of anti-inflammatory Th2 and Treg populations (Di Nicola et al., 2002; Aggarwal and Pittenger, 2005).

## MSCs and Atopic Dermatitis

Over the last few years, the immunomodulatory effect of MSCs-based therapy has been described in animal models and in human beings, showing a significant improvement in the clinical presentation by inhibiting the activation of T and B cells and, consequently, the release of anti-inflammatory cytokines (IL-10 and TGF-β), by decreasing the proliferation of IL-4 and IFNγ, and by decreasing the production of lgE (Dias et al., 2019).

Although several studies have demonstrated that the allergic progress in AD could be suppressed by MSCs derived from human umbilical cord blood (UCB-MSC), bone marrow (BMMSC) or adipose tissue (AD-MSC) by modulating multiple targets, there are some important issues to be considered in the stem cell-based therapy, such as the stem cell type used, number of cells transplanted, preconditioning of the cell preparation, relevant targets of the therapy, route and frequencyofadministration (Na et al., 2014; Kim et al., 2015; Shin et al., 2017b; Kim D. S. et al., 2018).

Human umbilical cord-derived mesenchymal stem cells (hUCB-MSCs) produced a significant protective and therapeutic effect against *Dermatophagoides farinae* (*Df*)-induced AD in mice by inhibiting MC degranulation. The protective effect was observed more prominently when local subcutaneous (SC) injection of MSCs was performed, when compared with intravenous (IV) administration, showing considerably importance in choosing the route of administration (Shin et al., 2017b). In another study, two different doses (low dose: 2 × 10^5^; high dose: 2 × 10^6^) of human adipose-derived mesenchymal stem cells (hAD-MSCs) were compared in a *Df*-induced mouse AD model (Shin et al., 2017a). The hAD-MSCs were injected intravenously in this study and interestingly, the higher dose of hAD-MSCs significantly reduced the clinical severity of AD in mice compared to the low dose group.

Another example of the complexity of stem cell-based therapy was demonstrated in a study in which hUCB-MSCs were pre-treated with MC granules, enhancing their therapeutic effects, as observed by the attenuation of AD signs in a NC/Nga mouse model. Moreover, it was shown that hUCB-MSCs primed with mast cell granules suppressed the activation of MCs and B lymphocytes more efficiently than naïve MSCs, both *in vitro* and *in vivo* (Lee et al., 2019).

However, the underlying mechanisms by which MSCs attenuate allergic responses is relatively unclear, considering that most studies have not focused on local, lesion specific therapeutic approaches, but rather on the regulation of systemic inflammatory responses (Kim et al., 2017). Accumulating data indicate that MSCs are not spontaneously immunosuppressive, but require stimulation for acquiring their immunomodulatory properties. In particular, the most important priming factors of MSCs are IFN-γ, TNF-α, and IL-1β. The release and binding of IFN-γ on its receptor expressed by MSCs are key steps for the induction of their immunomodulatory properties, not only for various T cell subtypes, but also against B and NK cells (Kim M. et al., 2018; Najar et al., 2018; Wobma et al., 2018). During the synergistic action of IFN-γ and TNF-α, an increased production of IL-6, IL-8, HGF, PGE_2_ and cyclooxygenase-2 (COX-2) was observed (Na et al., 2014; Lee and Song, 2018).

## Effects of MSCs on T Cells in the Context of AD

The pathogenesis of AD is mainly associated with T cell abnormalities, especially CD4^+^ T cells (Leung, 1999; Meagher et al., 2002). Based on the profile of cytokines produced, can be classified in Th1 or Th2 cells, and both cells play a critical role in AD pathogenesis (Hanifin and Chan, 1999; Leung, 1999). In the acute phase, Th2 response predominates with increased levels of several cytokines, including IL-4 and IL-13, which induce the production of IgE by B cells (Chaudhary et al., 2019). During the chronic phase, a predominance of Th1 profile has been described, with increased production of IL-5, IL-12, and IFN-γ, the latter being responsible for inhibiting Th2 lymphocytes (Leung, 1999). Therefore, due to the central role of Th1/Th2 balance in the pathogenesis of AD, any therapy able to modulate these profiles may potentially interfere with the evolution of the disease (Boothe et al., 2017).

Interestingly, MSCs present strong immunomodulatory effects on lymphocyte function (Di Nicola et al., 2002; Aggarwal and Pittenger, 2005; Batorov et al., 2015; Li et al., 2016), including immunomodulatory effects shown in AD models (Table 1). In BALB/c mice with AD induced by ovalbumin (OVA), treatment with superoxide dismutase 3-transduced MSCs (SOD3-MSCs) suppressed the recruitment of T cells into the skin and reduced the number of CD4^+^ and CD8^+^ T cells in the spleen and lymph node (Sah et al., 2018). A similar inhibitory effect on T cell recruitment to the skin was observed in BALB/c mice with AD induced by *Aspergillus fumigatus* (*Af*) and treated with hUCB-MSCs primed with poly I:C or IFN-γ and in NC/Nga mice with AD induced by *Df* and treated with hUCB-MSCs pretreated with MC granules (Lee et al., 2019; Park et al., 2019).

**TABLE 1:** Effect of MSCs on experimental animal models of atopic dermatitis.

Model	Animals (strain)	MSCs				
		
		Source	Route	Effect	Mechanism and note	References
–	Dogs	cAD-MSCs	IV	N	Systemic administration of cADMSCs appears safe but ineffective	Hall et al., 2010
AD (OVA-induced)	Mouse (BALB/c or C3H/HeN)	BM-MSCs	IV	Y	T-cell supression via NO; B cell supression via CSR	Na et al., 2014
AD (*Df*-induced)	Mouse (Nc/Nga)	hUCB-MSCs	SC	Y	Inhibition of MC degranulation throught PG2 and TGFβ1	Kim et al., 2015
AD (*Df*-induced)	Mouse (Nc/Nga)	hAD-MSCs	IV	Y	B cell supresion via (COX)-2	Shin et al., 2017b
AD (DNCB-induced)	Mouse (BALB/c)	hAD-MSCs	IV	Y	Regulating the expression of MIP-2, miR-122a-SOCS1, and Th1/Th2 responses	Kim M. et al., 2018
AD (OVA-induced)	Mouse (BALB/c)	hSOD3-MSCs	SC	Y	Suppression of response elicited by keratinocytes, mast cells, neutrophils, DCs, and T cells through multiple mechanisms	Sah et al., 2018
–	Dogs	cAD-MSCs	IV	Y	Supression of canine PBMC proliferation	Villatoro et al., 2018
AD (*Df*-induced)	Mouse (Nc/Nga)	hUCB-MSCs	SC	Y	Preconditionng of MSC with MC granules optimizes the supression of MC and B cells	Lee et al., 2019
AD (*Af*-induced)	Mouse (BALB/c)	hUCB-MSCs	SC	Y	Control both eosinophil-associated Th2 immunity and neutrophil-related Th17	Park et al., 2019

**AD, Atopic dermatitis; OVA, ovalbmin; Df, Dermatophagoides farinae; DNCB, dinitrochlorobenzene; Af, Aspergillus fumigatus; cAD-MSCs, canine adipose-derived mesenchymal stem cells; BM-MSCs, bone marrow-derived mesenchymal stem cells; hUCB-MSCs, human umbilical cord blood mesenchymal stem cells; hAD-MSCs, human adipose-derived mesenchymal stem cells; hSOD3-MSCs, human superoxide dismutase 3- transduced mesenchymal stem cells; IV, intravenous route; SB, subcutaneous route; NO, nitric oxide; CSR, class switch DNA recombination; MC, mast cell; PGE_2_, prostaglandin E_2_; COX, cyclooxygenase; MIP-2, macrophage inflammatory protein 2; SOCS1, suppressor of cytokine signaling 1; Th1, T helper 1; Th2, T helper 2; DC, dendritic cells; PBMC, peripheral blood mononuclear cell; Th17, T helper 17.**

*In vivo* findings of T cell inhibition in AD are supported by *in vitro* experiments in which co-cultures of MSCs with T lymphocytes showed suppression of T-cell proliferation and cytokine (IFNγ and IL-4) production (Na et al., 2014). Moreover, inhibition of T-bet and GATA-3 expression, which are transcription factors regulating IFNγ and IL-4 production, respectively, was also observed after treatment with BM-MSCs. Using L-NMMA, a nitric oxide (NO) inhibitor, the suppressive effect of BM-MSCS on T-cell proliferation and IFNγ production, but not IL-4 production, was reversed (Na et al., 2014).

## Effects of MSCs on B-Cells and IgE Production in the Context of AD

B cells play a critical role in the immune system and abnormalities on these cells functions result in a variety of chronic inflammation and autoimmune-mediated disorders, including AD (Simon et al., 2008; Nagel et al., 2009). MSCs are well known as suppressors of B cell function (Corcione et al., 2005; Asari et al., 2009; De Miguel et al., 2012) and, therefore, several studies evaluated their effect on B cells in AD models (Table 1).

Initially, Na et al. (2014) evaluated the effects of BM-MSC in BALB/c mice with AD induced by OVA. An intravenous injection of BM-MSCs was shown to suppress AD via inhibition of IgE production by B cells (Na et al., 2014). A reduction in IgE production in sera was also found in NC/Nga mice with AD induced by *Df* and treated with hAD-MSCs or hUCB-MSCs (Shin et al., 2017b; Lee et al., 2019).

*In vitro* experiments have confirmed the inhibitory effects of different types of MSCs on IgE production by B cells stimulated with lipopolysaccharide (LPS) or anti-CD40, as well as the inhibition of B cell proliferation and maturation (Na et al., 2014; Shin et al., 2017a; Lee et al., 2019). Interestingly, the effects of MSCs on B cell were attenuated with the addition of celecoxib, a selective COX-2 inhibitor (Shin et al., 2017b; Lee et al., 2019). In addition, BM-MSCs-induced IgE suppression is associated downregulation of activation-induced cytidine deaminase (AID) and B lymphocyte-induced maturation protein-1 (BLIMP-1), important regulators for class switch DNA recombination (CSR) and B-cell differentiation (Na et al., 2014). In short, we can see that MSCs modulate the maturation, proliferation and production of IgE, through CSR or COX-2-PGE_2_ pathway.

## Effects of MSCs on Mast Cells in the Context of AD

Mast cells regulate trafficking and functions of cells involved in the skin inflammatory response through the release of several soluble mediators, including chemokines, cytokines and growth factors (Liu et al., 2011). In AD, mast cells contribute to the pathogenesis of both acute and chronic lesions, and its presence is supported by higher concentrations of its products such as IL-4, IL-13 and histamine in AD patients (Ring and Thomas, 1989; Hamid et al., 1996; Liu et al., 2011). In addition, mast cell degranulation has been shown to correlate with AD severity (Zhao et al., 2006).

Remarkably, MSCs are able to inhibit mast cell degranulation in AD mouse models (Table 1; Kim et al., 2015; Kim M. et al., 2018; Sah et al., 2018; Lee et al., 2019; Park et al., 2019). In NC/Nga mice with AD induced by *Df*, hUCB-MSCs injected subcutaneously decreased the number of total degranulated mast cells, as well as the rate of degranulation (Kim et al., 2015; Lee et al., 2019). The inhibitory effect of MSCs on mast cell degranulation was also confirmed in BALB/c mice with AD induced by dinitrochlorobenzene (DNCB) and treated with hAD-MSCs and in BALB/c mice with AD induced by OVA and treated with SOD3-MSCs (Kim D. S. et al., 2018; Sah et al., 2018).

The inhibitory effect of MSC on mast cell degranulation was initially attributed to COX-2-PGE_2_ pathway (Kim et al., 2010). Previous reports revealed that human MC express multiple PGE_2_ receptors (EP receptors) that activate (EP_3_) or inhibit (EP_2_) mast cell degranulation (Feng et al., 2006; Kay et al., 2006; Wang and Lau, 2006). The addition of antagonists for the EP_2_ and EP_4_, but not for the EP_1_ and EP_3_, reduced the effect of hUCB-MSCs on mast cell degranulation (Kim et al., 2010). In agreement with this finding, MSCs treated with celecoxib, a selective COX-2 inhibitor, have a weak inhibitory effect on mast cell degranulation (Shin et al., 2017a; Lee et al., 2019). Taken together, the data support the involvement of COX-2-PGE_2_ pathway on MSC immunosuppression effect of mast cell degranulation.

In addition, Kim et al. (2015), using sirRNA, observed a loss in degranulation-inhibiting effect of MSCs with down-regulation of TGF-β1, which can be explained by the inhibitory effect of TGF-β1 produced on mast cells expression of high-affinity IgE receptor (FcεRI), a critical component for IgE-mediated degranulation (Gomez et al., 2005).

Another mechanism related to MSCs effects on mast cell degranulation is the reduction of reactive oxygen species (ROS) production, since ROS triggers mast cell activation through both FcεRI and histamine H_4_ receptor (H4R)-dependent pathways (Son et al., 2006; Swindle et al., 2008; Sah et al., 2018). Moreover, MSCs inhibit nuclear factor-kappa B (NFκB), which has been reported to bind to the H4R promoter region and, thereby, drive H4R upregulation and activation (Cogé et al., 2001).

Due to the promising profile of different types of MSCs to treat AD, several strategies to improve the inhibitory effects of MSCs in mast cell degranulation and on other immune cells are in process of development. These include the use of MSCs treated with muramyl dipeptide (stimulus for NOD activation) (Kim et al., 2015), MSCs genetically modified to overexpress superoxide dismutase 3 (Sah et al., 2018), hUCB-MSC primed with poly I:C or IFN-γ (Park et al., 2019) and MSCs preconditioning with mast cell granules (Lee et al., 2019). All these strategies were shown to improve the inhibitory effects of MSCs on mast cell degranulation, as well as in other immune cells, such as B and T cells. In addition, extracts from hUCB-MSC and exosomes derived from hAD-MSC also ameliorated AD, reinforcing the importance of the paracrine effects of MSCs in AD context (Cho et al., 2018; Song et al., 2019).

## Clinical Trials of MSCs Therapy in AD

Despite the evidence indicating the benefits of MSCs in the treatment of AD in pre-clinical studies, to the date of this review, only one clinical trial (phase I/IIa) was published with subjects with AD (Table 2). A single cell administration hUCB-MSCs subcutaneously was performed in 34 adult participants with moderate-to-severe AD. Using the eczema area and severity index (EASI) score, an improvement in AD symptoms was observed in the groups treated with the two doses (2.5 × 10^7^cells, low dose; 5 × 10^7^ cells, high dose) tested. In particular, the group treated with the higher dose of hUCB-MSCs showed a 50% reduction in EASI score in 6 of 11 (55%) subjects, without the appearance of side effects. In addition, the serum IgE levels and the number of eosinophils, typical biomarkers of AD, also decreased after treatment (Kim et al., 2017). Despite the encouraging results, this study has limitations, such as the small number of participants, its open label design and the lack of a placebo group. New studies with an experimental design including placebo groups and a larger number of patients, which are ongoing or to be started (Table 2), may bring new data to help define the clinical future of MSCs therapy in AD (National Library of Medicine, 2020).

**TABLE 2:** Clinical trials of MSCs in AD.

Type	Participants	Stem cells source	Route of administration	Result	References
Phase I; Phase IIa	7 adults; 27 adults	hUCB-MSCs	Subcutaneous	↓ IgE levels ↓ Neutrophil number 6/11 (55%): EASI50 in high dose treated group	Kim et al., 2016
Phase I	13 adults	MSCs	Intravenous	Ongoing	**NCT02888704**
Phase II	118 adults	hAD-MSCs	Intravenous	Recruiting	**NCT04137562**
Phase I; Phase II	20 adults; 72 adults	hBM-MSCs	Intravenous	Not yet recruiting	**NCT04179760**

*EASI, Eczema Area and Severity Index; eczema hUCB-MSCs, human umbilical cord blood mesenchymal stem cells; hAD-MSCs, Human adipose tissue mesenchymal stem cells; hBM-MSCs, human bone marrow mesenchymal stem cells. NCT; Identification code given to each clinical study registered on ClinicalTrials.gov.*

## Conclusion

Atopic dermatitis has become a significant public health problem due to its increasing prevalence, and there is a need for new therapeutic options for this disease. Evidence of therapeutic efficacy and mechanisms of action produced in pre-clinical studies indicate that MSC-based cell therapy is a promising approach for the treatment of AD. There is a need, however, for the conduction of double-blinded, placebo-controlled studies, to indicate the potential clinical application of MSCs, especially taking into account the complex pathogenesis of AD. Additional studies aiming at uncovering the mechanisms of action of MSC in atopic dermatitis may help define better therapeutic strategies for this disease.

## Author Contributions

SD and CM designed the study and wrote the manuscript. SD and IS conceived the artwork and performed the bibliographical research. RR and MS supervised the writing. All the authors revised and approved the final version of the manuscript.

## Conflict of Interest

The authors declare that the research was conducted in the absence of any commercial or financial relationships that could be construed as a potential conflict of interest.
